# Zika Virus Circulation in Mali

**DOI:** 10.3201/eid2605.191383

**Published:** 2020-05

**Authors:** Issa Diarra, Elif Nurtop, Abdoul Karim Sangaré, Issaka Sagara, Boris Pastorino, Souleymane Sacko, Amatigué Zeguimé, Drissa Coulibaly, Bakary Fofana, Pierre Gallian, Stephane Priet, Jan Felix Drexler, Anna-Bella Failloux, Abdoulaye Dabo, Mahamadou Ali Thera, Abdoulaye Djimdé, Bourèma Kouriba, Simon Cauchemez, Xavier de Lamballerie, Nathanaël Hozé, Ogobara K. Doumbo

**Affiliations:** Unité des Virus Émergents, (UVE: Aix-Marseille Univ-IRD 190-INSERM 1207-IHU Méditerranée Infection), Marseille, France (I. Diarra, E. Nurtop, B. Pastorino, P. Gallian, S. Priet, X. de Lamballerie);; Malaria Research and Training Center—Université des Sciences, des Techniques et des Technologies de Bamako, Mali (I. Diarra, A.K. Sangaré, I. Sagara, A. Zeguimé, D. Coulibaly, B. Fofana, A. Dabo, M.A. Thera, A. Djimdé, B. Kouriba, O.K. Doumbo);; Centre d’Infectiologie Charles Mérieux, Bamako (A.K. Sangaré, B. Kouriba);; Ministère de la Santé et des Affaires Sociales du Mali, Bamako (S. Sacko);; Laboratoire de Virologie, Établissement Français du Sang Provence-Alpes Côte-d'Azur et Corse (EFS), Marseille (P. Gallian);; German Center for Infection Research, Bonn-Cologne, Germany (J.F. Drexler);; Charité-Universitätsmedizin Berlin, Institute of Virology, Berlin, Germany (J.F. Drexler);; Institut Pasteur, Paris, France (A.-B. Failloux, S. Cauchemez, N. Hozé);; Centre National de la Recherche Scientifique, Paris (S. Cauchemez, N. Hozé)

**Keywords:** Zika virus, seroprevalence, Mali, viruses, arbovirus, vector-borne infections, mosquitoes

## Abstract

The circulation of Zika virus (ZIKV) in Mali has not been clearly characterized. Therefore, we conducted a serologic survey of 793 asymptomatic volunteers >15 years of age (2016), and 637 blood donors (2013) to assess the seroprevalence of ZIKV infection in 2 ecoclimatic regions of Mali, tropical savannah and warm semiarid region, using ELISA and seroneutralization assays. The overall seroprevalence was ≈12% and increased with age, with no statistical difference between male and female participants. In the warm semiarid study sites we detected immunological markers of an outbreak that occurred in the late 1990s in 18% (95% CI 13%–23%) of participants. In tropical savannah sites, we estimated a low rate of endemic transmission, with 2.5% (95% CI 2.0%–3.1%) of population infected by ZIKV annually. These data demonstrate the circulation of ZIKV in Mali and provide evidence of a previously unidentified outbreak that occurred in the late 1990s.

Zika virus (ZIKV) is an arbovirus (genus *Flavivirus*; enveloped positive-stranded RNA virus) (*1*). The isolation of ZIKV took place in 1947 from a caged sentinel rhesus monkey during a yellow fever virus survey conducted in Zika forest of Uganda. The first notable human epidemic was recorded on the Western Pacific Islands of Yap, Federated States of Micronesia, in 2007 (*2*,*3*). The Asian genotype of the virus spread in the Pacific Islands, then to Latin America and the Caribbean (*4*).

ZIKV is transmitted to humans primarily through the bite of *Aedes* spp. mosquitoes; however, sexual and maternofetal routes of transmission have been identified during recent outbreaks (*5*–*7*). ZIKV infections are believed to be frequently asymptomatic or pauci-symptomatic (common mild and self-limiting symptoms that include macular or papular rash, fever, arthritis, conjunctivitis and headache) (*8*). In Martinique, estimates of truly asymptomatic cases among blood donors infected by ZIKV were ≈45%, and cases that did not require medical attention were ≈80% (*9*). A range of 29%-82% for asymptomatic infections has been reported (*10*), possibly reflecting differences between human populations or viral strains but most likely because of the imprecise nature of the definition of asymptomatic cases. However, recent outbreaks in Polynesia and the Americas have shown rare but serious complications, such as eye lesions (*11*), neurologic conditions in adults (myelitis, encephalitis, Guillain-Barré syndrome) (*12*) and developmental abnormalities (including microcephaly) in fetuses (*13*).

Although the first identification of ZIKV was made in Africa, Zika virus disease did not draw much attention in Africa, possibly because no large outbreaks were detected that were caused by the African genotype of the virus, or because it was misdiagnosed as a generic arboviral febrile illness. Reports of ZIKV isolation from humans and mosquitoes, as well as several seroepidemiological surveys, showed that the virus has been circulating endemically in countries in Africa for decades (*14*,*15*). The reason is the sylvatic transmission cycle of the African genotype, which differs from the recently observed urban transmission cycle of the Asian genotype (*16*,*17*).

The risk for epidemic spread of ZIKV in Africa is a public health issue. Strains belonging to the African genotype may over time become adapted to peridomestic mosquitoes circulating in Africa and cause large urban epidemics (*18*). Furthermore, recent epidemics in Cabo Verde (*19*) and Angola (*20*) are reminders that the importation of ZIKV strains belonging to the Asian genotype and adapted to urban mosquitoes may cause the spread of the virus in mostly naïve populations.

We present the results of a cross-sectional seroepidemiological study and report evidence of ZIKV circulation in Mali. Our work is part of a global effort to characterize more accurately the circulation of ZIKV in Africa, estimate the level of immunity of local populations, and identify populations vulnerable to future epidemics.

## Methods

### Study Design and Population

We conducted a cross-sectional study primarily dedicated to establishing the seroprevalence of arboviral and hemorrhagic viral infections in Mali during October–November 2016 in 7 sites. In Bamako only, we also used 637 serum samples collected in 2013 from eligible volunteer blood donors for studying the seroprevalence of arboviral infections.

The Institutional Review Board of the Faculty of Medicine and Odonto-Stomatology, University of Sciences, Techniques and Technologies, Bamako, Mali, reviewed and approved this study (IRB letter no. 2016/113/CE/FMPOS). First, we visited all sites to meet health professionals, administrative authorities, and local community to explain the study context; after obtaining community permission, the study staff visited participant families. We conducted the study in alignment with institutional procedures and guidelines.

**Figure 1:**
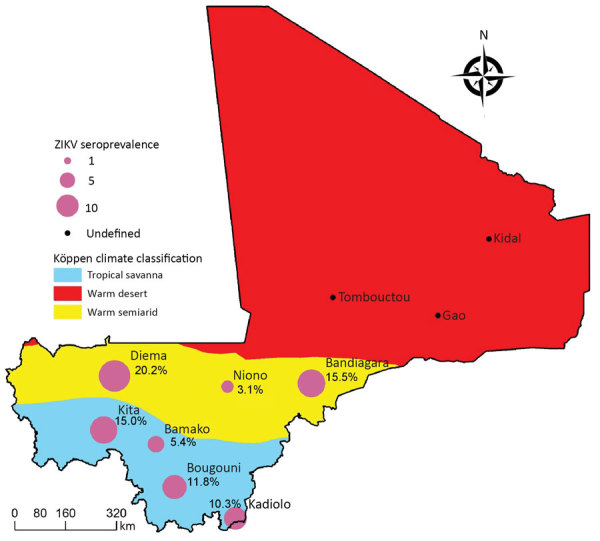
The main climatic zones of Mali by Köppen climate classification and sites of study of Zika virus seroprevalence.

We selected 7 districts representing the different ecoclimatic areas of Mali: Diéma, Kita, Bougouni, Kadiolo, Niono, Bandiagara, and Commune IV of Bamako (Figure 1). Those districts are spread over the different administrative regions of Mali, excluding the northern region, which could not be investigated for security reasons. The selected districts are also those used by the Ministry of Health for infectious diseases surveillance.

We selected villages or city areas randomly from an exhaustive list in each district. We randomly selected families and recruited volunteers with the objective of including 100–150 participants per site (Table 1), which corresponds to the estimate of the recruitment capacity by field teams for each site investigated. This sample size allows establishing seroprevalence in each site and globally. For significance level α = 0.05 and seroprevalence values 5%–30%, the precision varies from ≈4% to 9% (sample size = 100), and from ≈1.5% to 3% (sample size = 800).

**Table 1:** Precision of seroprevalence determination according to prevalence estimates and sample size in study of Zika virus, Mali*

Prevalence estimate	Precision for sample size		
	100	150	800
0.050	0.044	0.035	0.015
0.100	0.060	0.048	0.020
0.150	0.070	0.058	0.025
0.200	0.080	0.065	0.028
0.250	0.086	0.070	0.030
0.300	0.091	0.074	0.032

*α = 0.05.

We enrolled only healthy, nonfebrile male and female volunteers >15 years of age. We provided study information in the most familiar language of the volunteer and in the presence of an observer designated by the village authorities who ensured that volunteers understood the information completely and helped obtain answers to any question. All volunteers signed the informed consent form before enrollment and received a copy of the signed form.

We used the Open Data Kit (ODK) platform on tablets to collect data, including sociodemographic information, weight, and history of travels. The same day, we drew venous blood (5 mL) in SST vacutainer tubes (Becton Dickinson, https:/www.bd.com), kept them at 2°C –8°C, and centrifuged (1,500–2,000 × *g*) within 12 h before aliquoting and storing at −80°C.

Eligibility criteria for blood donors in Bamako included acceptance and signature of the consent form, age (18–55 y), a normal blood pressure, a bodyweight >50 kg, the absence of recent fever or history of fever, and negative testing for HIV, hepatitis B and C, and syphilis. We excluded persons with known chronic disease or sensitivity to blood collection or those who were pregnant or breastfeeding. We heat inactivated samples collected in Mali, packed them at the Malaria Research and Training Center in Bamako, and sent them to the National Reference Centre for Arboviruses (Unité des Virus Émergents, Marseille, France) for serologic investigations, in compliance with national regulations in both countries.

### Serologic Analysis

Before the analysis, we inactivated serum specimens at 56°C for 30 min. We detected ZIKV IgG using a primary ELISA screening (Euroimmun anti-NS1 IgG ELISA kit, Medizinische Labordiagnostika, https://www.euroimmun.com); the sensitivity of the ELISA appears to be high in these studies (*21*). We further investigated serum samples that yielded a positive or equivocal ELISA result using a cytopathic effect (CPE)–based virus neutralization test (VNT) and a strategy previously described and validated for seroprevalence studies. Serum samples with a neutralizing titer >40 were considered positive. This strategy enables us to reach sensitivity of 98.1% and specificity of 98.8% in a population with high exposure to dengue virus infection (*22*).

### Statistical Analyses

We used IBM SPSS Statistics 24 (http://www.ibm.com) to perform statistical analyses. We evaluated associations between sociodemographic variables (age and sex) and ZIKV seroprevalence by logistic regression and Pearson χ^2^ test.

The analysis of seroprevalence stratified by age can provide insight on the history and mode of circulation of a pathogen (*23*). For example, a slow continuous rise of seroprevalence with age may indicate endemic circulation, whereas a sudden increase in seroprevalence for persons >20 years of age suggests that an outbreak took place 20 years ago. We used serocatalytic statistical models to reconstruct the force of infection, defined as the per capita rate of infection of susceptible persons, from such data (*23*).

We considered different statistical models; in the constant model, we assumed that the force of infection λ is constant with age and time. In that case, the seroprevalence of an individual is expected to increase with their age *a*: *Pa* = 1 − exp(−λ.*a*). In the epidemic model, we assume that infections occurred during an epidemic. The force of infection was equal to during the epidemic year and 0 at other times. Persons born after the epidemic were therefore all seronegative and older ones had a probability p = 1 − exp(−λ) of being seropositive.

We fitted these statistical models to data with a Markov Chain Monte Carlo (MCMC) Metropolis-Hastings sampler implemented in the Rstan package (*24*). We chose flat priors for the parameters and simulated 4 independent chains of 5,000 runs (2,500 burn-ins) for each fit. For the constant model, we reported the mean and 95% credible interval of the annual probability of infection of a susceptible patient using the formula *PI* = 1 − exp(−λ.*a*). The deviance information criterion (DIC) was used to assess the model fits (*25*). Lower DICs indicate better fits, with a DIC difference of 5 considered substantial.

We grouped the localities according to local climate areas from Köppen classification: the cities of Diéma, Bandiagara, and Niono comprised a semiarid area, and the cities of Bamako, Kadiolo, Bougouni, and Kita comprised a tropical area. We fitted the models to each of these areas (Figure 1).

## Results

We included a total of 1,430 healthy volunteers. Overall, 793 healthy participants >15 years of age were enrolled in October–November 2016 in 7 different sites in Mali: Niono (n = 65), Bamako (n = 129), Kadiolo (n = 136), Bougouni (n = 127), Kita (n = 40), Bandiagara (n = 187), and Diéma (n = 109). The sex ratio (M/F) was 0.44 (242/551) and median age was 33 years. We also included serum samples from 637 eligible blood donors from Bamako who provided samples in 2013. In this group, there were few women (sex ratio 8.8 (572/65) as previously observed in Mali blood donor populations (*26*), and the median age was 28.0 years (Table 2).

**Table 2:** Demographic characteristics of the population in study of Zika virus, Mali

Characteristic	Niono 2016, n = 65	Bamako		Kadiolo 2016, n = 136	Bougouni 2016, n = 127	Kita 2016, n = 40	Bandiagara 2016, n = 187	Diéma 2016, n = 109	Total, n = 1,430
		2016, n = 129	2013,* n = 637						
Sex									
M	9	50	572	40	42	16	58	27	814
F	56	79	65	96	85	24	129	82	616
M/F ratio	0.2	0.6	8.8	0.4	0.5	0.7	0.4	0.3	1.3
Median age, y	35	32	28	30	45	23	35	28	30

*Volunteer blood donors.

### ZIKV Seroprevalence

Using the ELISA+CPE-based VNT strategy, we found IgG seropositivity for ZIKV of 11.98% among the 793 serum specimens collected from participants in 2016. Seropositivity range was 3.1%–20.2% in different regions: 3.1% in Niono, 5.4% in Bamako, 10.3% in Kadiolo, 11.8% in Bougouni, 15% in Kita, 15.5% in Bandiagara, and 20.2% in Diéma (Table 3). The endpoint titer average was 26.32 + 5.41 (range 10–320). In a global analysis, we detected no statistical difference between sexes (11.6% in male participants, 12.2% in female participants; Pearson p = 0.834); however, in Bougouni the seroprevalence was significantly higher in male participants (21.4% vs. 7.1% in female participants; p = 0.04).

**Table 3:** Results of seroepidemiological investigations for Zika virus according to study sites and time of sampling, Mali

Study site and year	Total no.	IgG* doubtful, no. (%)	IgG* positive, no. (%)	VNT† positive, no. (%)
Niono 2016	65	4 (6 0.2)	4 (6.2)	2 (3.1)
Bamako 2016	129	0 (0.0)	11 (8.5)	7 (5.4)
Bamako 2013‡	637	18 (2.8)	67 (10.5)	47 (7.4)
Kadiolo 2016	136	14 (10.3)	22 (16.2)	14 (10.3)
Bougouni 2016	127	3 (2.4)	22 (17.3)	15 (11.8)
Kita 2016	40	5 (12.5)	7 (17.5)	6 (15.0)
Bandiagara 2016	187	24 (12.8)	81 (43.3)	29 (15.5)
Diéma 2016	109	14 (12.8)	39 (35.8)	22 (20.2)
Total	1,430	82 (5.7)	253 (17.7)	142 (9.9)

*Result by Euroimmun IgG ELISA assay. †Result by cytopathic effect-based virus neutralization test. ‡Volunteer blood donors.

In blood donors sampled in 2013, seroprevalence was 7.4% (47/637), and we found no statistical difference between male and female participants (7.9% versus 3.1%, Pearson p = 0.162). The seroprevalence values in blood donors sampled in 2013 and participants of commune IV of Bamako sampled in 2016 were similar: 7.4% (47/637) from 2013 versus 5.4% (7/129) from 2016 (Pearson p = 0.430). This finding suggests the absence of sustained ZIKV circulation in this area between 2013 and 2016, making it possible to pool results obtained in Bamako for the estimation of the force of infection.

**Figure 2:**
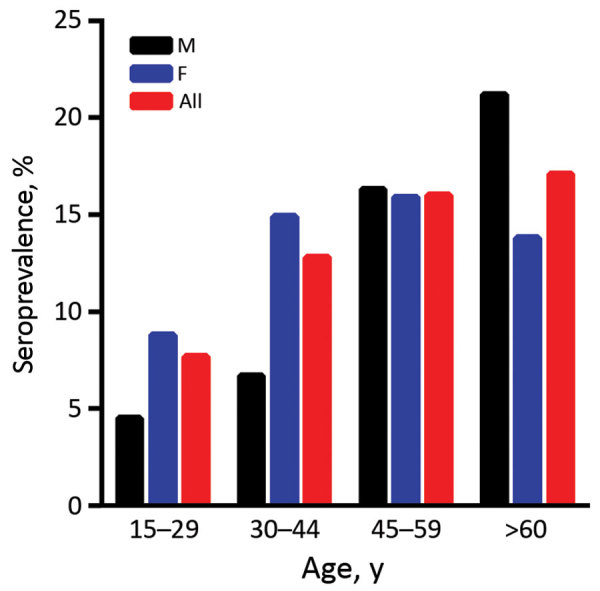
Zika virus seroprevalence by age group, Mali, 2016.

When we categorized the 2016 cross-sectional study population into age groups, we found that ZIKV seropositivity increases with age (logistic regression p = 0.003, 95% CI 1.006–1.029): 7.7% for the 15–29-year age group, 12.8% for 30–44 years, 16.0% for 45–59 years, and 17.1% for >60 years (Figure 2). This trend was maintained when we included the blood donors population (7.3% for the 15–29-year age group, 9.6% for 30–44 years, 14.6% for 45–59 years, and 17.1% for >60 years; p = 0.000, 95% CI 1.011–1.031). Seventy of the 793 participants of 2016 reported travels during the 6 months preceding blood collection, including 7 who VNT-ZIKV positives, and 88 VNT-ZIKV positives of the remaining 723 participants did not report travel (no statistical difference; Pearson p = 0.593).

### ZIKV Transmission Dynamics

**Figure 3:**
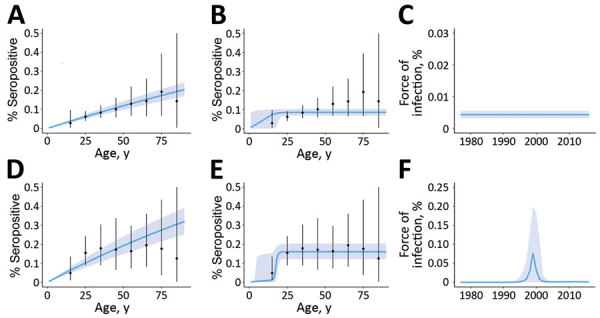
Observed and predicted profiles for Zika virus seroprevalence by age, climatic zone, and the assumed mode of transmission, Mali. Observed age-specific seroprevalence mean (black dots) and range (error bars) are compared with predictions (blue lines) of models; shading indicates 95% CI. Panels A–C show data for tropical savannah and D–F for semiarid regions. Predictions assume a constant force of infection over time (A, D) or a single epidemic in the past (B, E). Force of infection is shown over time by the best fitting model for each climatic region (C, F).

We found that the continuous increase of seroprevalence with age in the tropical savannah area was better explained by a model assuming constant low-level circulation of ZIKV (Figure 3, panel A) than one assuming a single outbreak occurred in the past (Figure 3, panel B). From the best fitting model with a constant force of infection, we estimated that 2.5% (95% CI 2.0%–3.1%) of the susceptible population is infected by ZIKV annually (Figure 3, panel C). In the semiarid areas, we saw seroprevalence sharply increase for participants >20 years of age and then stabilize (Figure 3, panels D, E). This age profile was best explained by the epidemic model, with an epidemic expected to have occurred in the late 1990s (Figure 3, panel F). The proportion of the population infected at that time was estimated to be 18% (95% CI 13%–23%).

## Discussion

We conducted a Zika seroepidemiological study in Mali and found evidence for the circulation of ZIKV. We found an average ZIKV seroprevalence of ≈12%, ranging from 3.1% to 20.2% depending on the study site. Seroprevalence was higher than expected because recent surveys conducted in several Central and West Africa countries reported lower values: ≈5% in Cameroon (*17*), 0.1% in the Democratic Republic of the Congo (*27*) and 3.4% in Nigeria (*28*). Those countries have ecoclimatic conditions that have been typically associated with the circulation of ZIKV and its implication in the transmission to humans by peridomestic mosquitoes (i.e., tropical forested areas with nonhuman primates and sylvatic *Aedes* spp. mosquitoes). The situation is different in Mali, where this sort of ecologic environment is uncommon and can be found only in some sites located in the southwestern part of the country. Mali includes a tropical savannah belt in the south (containing the study sites Kita, Bamako, Bougouni, and Kadiolo), and a warm semiarid region (Diéma, Niono, and Bandiagara). Further north are vast Sahelian regions that we could not investigate for security reasons. Of note, a single ZIKV serosurvey was conducted previously in Mali in 1964–1967 and reported prevalence as high as 52% with hemagglutination inhibition (HI) assay, a method which is highly susceptible to cross-reaction with related flaviviruses that cause high false positive rates (*29*,*30*).

Of interest, when modeling our data we concluded that the most probable mode of transmission in the tropical savannah region was nonepidemic and associated with low seroprevalence values, which is reminiscent of the low-rate endemic transmission that has been reported in Central Africa (Cameroon and Congo) (*17*,*27*) and West Africa (*28*,*31*). This transmission may correspond in part to sylvatic exposure. In contrast, our best model points to the occurrence of a large ZIKV epidemic in the warm semiarid regions in the late 1990s (Figure 3), with seroprevalence values reaching 20.2% in Diéma. It is likely that transmission was facilitated by a peridomestic mosquito species (most probably *Aedes aegypti*), which raises the possibility that the cause was an imported Asian genotype strain or an African genotype strain with an improved competence for peridomestic mosquitoes.

The identification of 2 different transmission dynamic patterns of ZIKV, combined with the high percentage of neutralizing antibodies in several study sites, deserves further investigation. No strain of ZIKV has been isolated or characterized by molecular methods in Mali to date, and direct detection of ZIKV in the samples we studied was not possible due to systematic heat inactivation. Implementing entomological surveys and the study of nonmalarial febrile patients in Mali is necessary to isolate, sequence, and genetically characterize the circulating ZIKV strains in Mali and characterize the putative enzootic maintenance cycle of ZIKV in the tropical savannah region. In addition, the recent report of an association between ZIKV IgG and microcephaly in Guinea-Bissau (*32*) suggests that comprehensive case-control studies of pregnant women and their infants with congenital neurologic abnormalities should be performed in Mali to clarify the possible implication of ZIKV.

This study had several limitations. First, the size of the population tested and the number of sites included in the study were limited by severe logistical and security constraints. Accordingly, the population studied is not fully representative of the Mali general population. However, our results provide a novel and credible picture of the circulation of ZIKV according to the sites and age classes. Second, the circulation of potentially cross-reacting flaviviruses in the region raises questions of detection specificity. Nevertheless, previous investigations conducted in a region where the population is heavily exposed to dengue virus showed that the testing strategy we used in our study is robust and highly specific (*22*). Third, the detection of neutralizing antibodies may underestimate the actual proportion of the population that was infected by ZIKV if a fraction of the infected persons lose antibodies to ZIKV over time (*33*). This possibility requires further investigation to better understand the potential effects on seroprevalence studies.

In conclusion, we demonstrated the circulation of ZIKV in Mali, and observed seroprevalence rates that most probably are insufficient to create protective herd immunity against potential future outbreaks. The identification of a previously unknown ZIKV outbreak in the semiarid regions of Mali in the late 1990s emphasizes the need for improving the detection of emerging infections in Africa (*34*). Additional research revealing the dynamics of transmission, prevalence of symptomatic and asymptomatic ZIKV infections, and the frequency and severity of ZIKV-related congenital anomalies and other neurologic complications should be implemented.
